# Computational workflow for analysis of gain and loss of genes in distantly related genomes

**DOI:** 10.1186/1471-2105-13-S15-S5

**Published:** 2012-09-11

**Authors:** Andrey Ptitsyn, Leonid L Moroz

**Affiliations:** 1 Whitney Laboratory for Marine Biosciences, University of Florida; 9505 Ocean Shore Blvd. Saint Augustine FL 32080, USA; 2 Dept of Neuroscience, University of Florida; Gainesville, FL 32610, USA

## Abstract

**Background:**

Early evolution of animals led to profound changes in body plan organization, symmetry and the rise of tissue complexity including formation of muscular and nervous systems. This process was associated with massive restructuring of animal genomes as well as deletion, acquisition and rapid differentiation of genes from a common metazoan ancestor. Here, we present a simple but efficient workflow for elucidation of gene gain and gene loss within major branches of the animal kingdom.

**Methods:**

We have designed a pipeline of sequence comparison, clustering and functional annotation using 12 major phyla as illustrative examples. Specifically, for the input we used sets of *ab initio *predicted gene models from the genomes of six bilaterians, three basal metazoans (Cnidaria, Placozoa, Porifera), two unicellular eukaryotes (*Monosiga *and *Capsospora*) and the green plant *Arabidopsis *as an out-group. Due to the large amounts of data the software required a high-performance Linux cluster. The final results can be imported into standard spreadsheet analysis software and queried for the numbers and specific sets of genes absent in specific genomes, uniquely present or shared among different taxons.

**Results and conclusions:**

The developed software is open source and available free of charge on Open Source principles. It allows the user to address a number of specific questions regarding gene gain and gene loss in particular genomes, and user-defined groups of genomes can be formulated in a type of logical expression. For example, our analysis of 12 sequenced genomes indicated that these genomes possess at least 90,000 unique genes and gene families, suggesting enormous diversity of the genome repertoire in the animal kingdom. Approximately 9% of these gene families are shared universally (homologous) among all genomes, 53% are unique to specific taxa, and the rest are shared between two or more distantly related genomes.

## Introduction

In the past, a number of alternative approaches have been developed to determine evolutionary relationships between genomes and taxons. Clustering orthologous groups (COGs) on the basis of protein similarity [1-4] addresses the challenge of reconstructing the evolutionary tree from a set of related genes that behave in a semi-independent way, i.e. experience duplication, differentiation and extinction within both the same genome (paralogous) and differentiating genomes (orthologous) lineages. Some recently developed approaches like EvolMap [5], CAFE [6] or BadiRate [7] combine similarity scoring with pre-existing tree structure, which allows introduction of traditional morphology-based classification. These advanced methods are remarkably precise and effective in reconstruction of ancestral gene families and estimation of time since branching events in genome evolution.

However, the volume of data and high diversity in the gene composition present computational and interpretational challenges. A fast analysis of gene gain and loss in overall composition of genomes is particularly effective for resolving relations between distant taxons. Genes found both in a more basally branching lineage and a more derived lineage but having no homolog in an intermediately derived taxon may be lost there either through deletion or profound diversification. The challenge is to efficiently catalog all genes present in all, some or just one of the representative genomes. Here, we propose a workflow model for analysis of gain and loss of genes in distantly related genomes that can handle large data sets and produce reasonable results even beginning from rough draft genomes. To demonstrate applicability of the developed workflow we estimate the degree of gene gain and gene loss across 12 genomes representing key transitions in the evolution of multicellularity and rise of animal organization. Since more diverse invertebrate genomes are scheduled for sequencing in the near future, the pipeline is open to addition of any number of new genomes. As a result, these data would be important to elucidate the major events in genome organization linked to both genome-wide and more targeted molecular innovations within specific taxonomical groups.

## Methods

### Input data

For our study we have selected sequenced genomes of several representative animal phyla from basal Metazoans and some bilaterians: *Nematostella *(Cnidaria); *Trichoplax *(Placozoa); *Amphimedon *(Porifera), two protostomes such as *Daphnia *(Arthropoda) and *Lottia *(Mollusca); and four Deuterostomes such as *Strongylocentrotus *(Echinodermata), *Saccoglossus *(Hemochordata), *Homo *and *Branchiostoma *(Chordata). We have also selected single-cell eukaryotic genomes of *Monosiga *and *Capsaspora *representing potential sister taxa and a plant genome of *Arabidopsis thaliana *as an out-group. The sources and sizes of data are outlined in Table 1.

**Table 1:** Initial data for the analysis of gain and loss of genes in distant phyla of the animal kingdom.

Species	Source URL	Number of models	Input data size (MB)
*Arabidopsis thaliana*	ftp://ftp.arabidopsis.org/home/tair/home/tair/	27416	14.72
*Amphimedon queenslandica*	http://getentry.ddbj.nig.ac.jp/top-e.html?ACUQ00000000	30060	11.87
*Branchiostoma floridae*	http://genome.jgi-psf.org/Brafl1/Brafl1.home.html	50817	24.34
*Capsaspora owczarzaki*	http://www.broadinstitute.org/annotation/genome/multicellularity_project/GenomeDescriptions.html	8792	6.2
*Daphnia pulex*	http://wfleabase.org/genome/Daphnia_pulex/	30810	10.94
*Homo sapiens*	http://www.gencodegenes.org/	87069	42.55
*Lottia gigantea*	http://genome.jgi-psf.org/Lotgi1/Lotgi1.home.html	23851	9.72
*Monosiga brevicollis*	http://genome.jgi-psf.org/Monbr1/Monbr1.home.html	9171	5.77
*Nematostella vectensis*	http://genome.jgi-psf.org/Nemve1/Nemve1.home.html	27273	9.76
*Saccoglossus kowalevskii*	ftp://ftp.hgsc.bcm.tmc.edu/pub/data/Skowalevskii/fasta	13149	8.14
*Trichoplax adhaerens*	http://genome.jgi-psf.org/Triad1/Triad1.home.html	27416	14.72
*Strongylocentrotus purpuratus*	http://www.spbase.org/SpBase/download/	42420	22.07

The analysis workflow is applicable to sets of genomes with different degrees of completion so long as a sufficient number of predicted gene models representing the exome can be derived. For the case study we have also selected rough draft genomes generated by short-read high-throughput technology. In these genomes gene models predicted *ab initio *are often short, fragmented and contaminated by translated non-protein coding fragments. To make the data more consistent we used all unfiltered gene models for all genomes, even in cases of finished genomes for which refined sets of protein-coding genes are available.

### Computational pipeline

**Figure 1:**
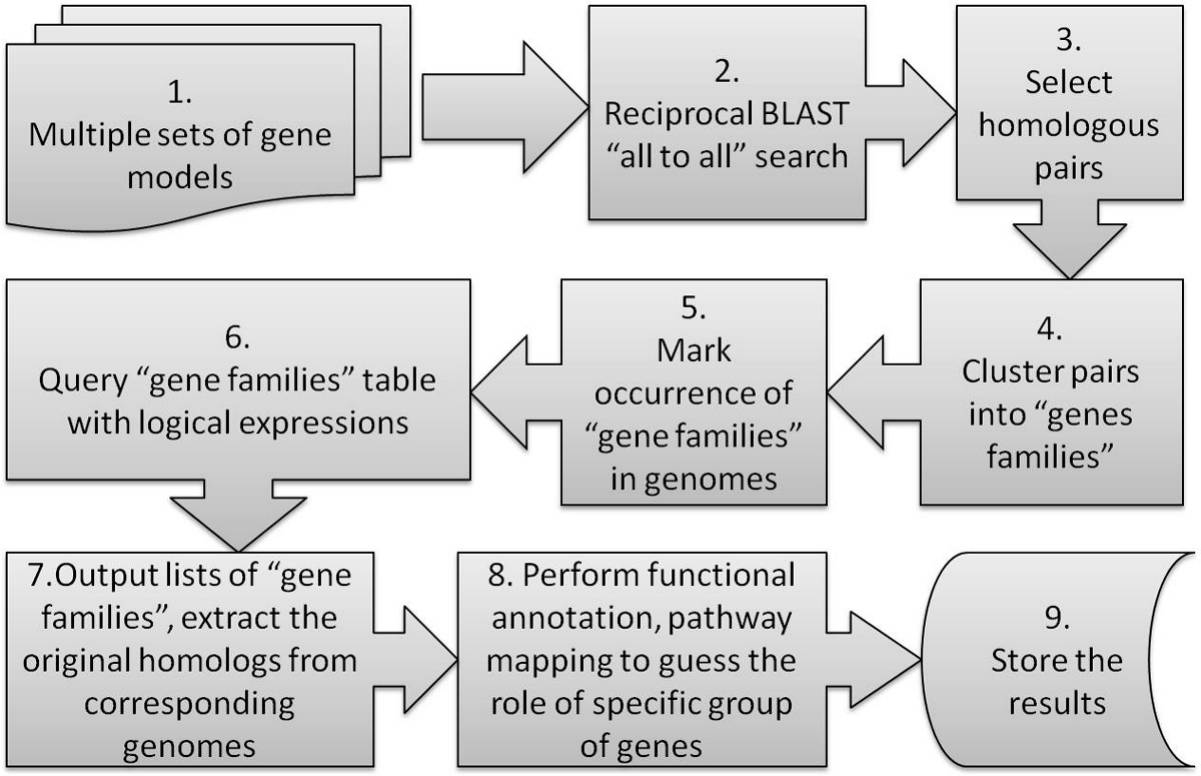
**Overview of the gene-gain and gene-loss analysis pipeline**. Steps 3, 4, 5 and 7 required new code development. Step 6 requires a Microsoft Excel spreadsheet or similar software.

Gene gain and gene loss in a group of distantly related genomes has been estimated by the workflow outlined in Figure 1. The initial input of our analysis pipeline consists of predicted protein models for a group of 12 selected genomes.

Each of the starting sets of gene models has been compared to itself using reciprocal BLAST[8]. All hits have been filtered to remove shorter sequences with high similarity (estimated by e-value equal to or lower than 0.0001). BLAST is faster than Smith-Waterman [8,9] used in other large-scale orthology delineation projects [10], yet still sufficiently sensitive to detect orthologous genes in large-scale analysis of distant genomes [11]. Resulting non-redundant sets of gene models have been compared to each other using reciprocal BLAST with the same similarity threshold. From the algorithmic point of view the result of this stage is transformation from the set of objects (gene models) to an adjacency graph connecting related gene models, while edges with similarity below a certain threshold are cut. Then pairs of similar sequences have been extracted from the tabulated BLAST output and clustered using greedy algorithm implemented in C. The algorithm iterates through the list of matching pairs marking connected objects as belonging to one cluster until no new objects are connected in a complete cycle.

The computationally selected broad collections of genes are mapped back on the original sets of gene models in the steps outlined in Figure 1. Here, we use the term "Animal Metagenome" to define the broadest formal category that refers to all recognized genes on a high taxonomic level of the animal kingdom. The results of our workflow are sets of names under which particular genes or gene families from the combined metagenome are found in individual genomes, and a table showing absence/presence of a particular gene in a given taxon. In our formal workflow any gene sets were added in alphabetic order, thus each gene homolog is tagged by the name under which it first appears. As a result, a single name can represent either a unique instance of a gene or a family of gene instances joined by clustering (see Figure 1.). The table of a given gene occurrence is a tab-delimited text file that can be imported in Excel or a similar spreadsheet application (see Table 2). The full table resulting from the model 12 genomes is given in supplementary materials (Additional File 1).

**Table 2:** Example of the final output (fragment) table imported in Excel spreadsheet.

gene	am	ar	br	ca	da	ho	lo	mo	ne	sa	tr	ur	sum	Bilateria	Basal
Aqu1.205579	1	1	1	0	0	0	0	1	1	0	1	0	7	TRUE	TRUE
Aqu1.208671	1	0	1	1	0	0	0	0	1	1	0	1	7	TRUE	TRUE
Aqu1.213232	1	1	1	0	0	1	0	0	0	1	1	1	8	TRUE	TRUE
Aqu1.219112	1	0	1	0	0	0	0	0	0	0	0	1	4	TRUE	TRUE
Aqu1.201795	1	0	0	1	0	0	0	0	0	1	0	1	5	TRUE	TRUE
Aqu1.227071	1	0	0	1	0	0	0	0	0	1	0	1	5	TRUE	TRUE

Rows represent selected genes (representative instances of gene families) from all Animal Metagenome pool, columns mark occurrence of the given gene from this pool in a particular genome. The last two columns have logical expressions for such gene occurrence in any Bilateria and in any Basal Metazoa genomes. Abbreviations: am -- *Amphimedon*; ar -- *Arabidopsis*; br -- *Branchiostoma*; ca -- *Capsaspora*; da -- *Daphnia*; ho -- *Homo*; lo -- *Lottia*; mo -- *Monosiga*; ne -- *Nematostella*; pb -- *Pleurobrachia*; tr -- *Trichoplax*; ur -- Sea urchin (*Strongylocentrotus*).

Any combination of genes present or absent in a given animal lineage can be imported in Excel and queried for genes uniquely shared among two or more genomes or absent in a particular genome, etc. For example, genes that occur in Bilateria are retrieved by expression OR (*Homo, Saccoglossus, Branchiostoma, Strongylocentrotus, Daphnia, Lottia*) where names correspond to Excel columns. Filtered subsets of any predicted gene gain-/loss combination from the "Animal Metagenome" can be exported and used for Pathway Analysis and Functional Annotation. Alternatively, the same subsets can also be saved as a list in a text file and then used to extract the original gene models from specific genomes for additional annotation ( e.g. homologs search in GenBank, various Pathway Analysis and Functional Annotation tools). We have also implemented a set of programs assisting these operations and used DAVID http://david.abcc.ncifcrf.gov/ for analysis of Gene Ontology, Protein Motif and KEGG pathway enrichment in selected sets of predicted gene gain or gene loss occurrences.

### Implementation and availability

The pipeline has been developed using a combination of existing software and new code in C. The open source software is available free of charge from the Whitney Laboratory website http://www.whitney.ufl.edu/PtitsynLab or from the authors by request. This work is licensed under a Creative Commons Attribution 3.0 Unported License: http://creativecommons.org/licenses/by/3.0/

## Results and discussion

**Figure 2:**
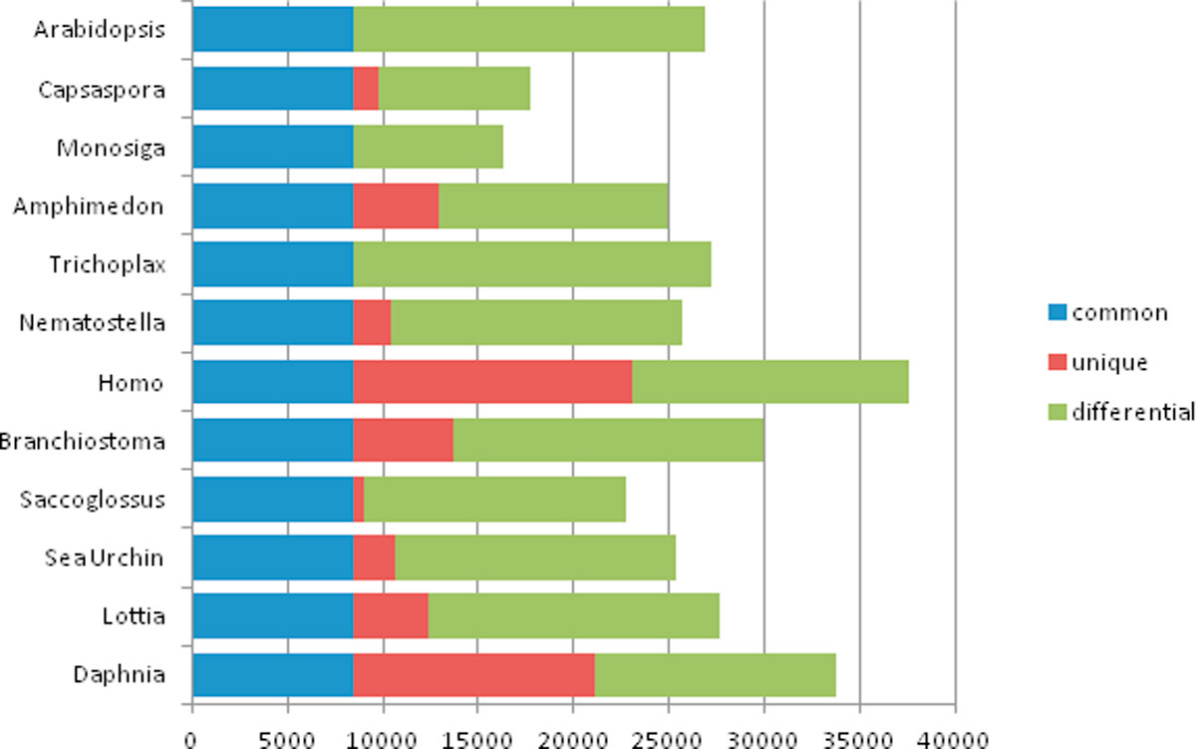
**Common, unique and differential occurrence of genes in major phyla**. Diagram shows absolute number of "all non-redundant genes from 12 genomes used" (horizontal axis) that map to a particular genome. Out of 92,320 total 8,501 gene families are found in all genomes and 52,910 are unique to a particular genome.

The combined dataset of even a relatively small fraction of the sequenced genomes revealed unprecedented diversity of gene families reflecting the extensive parallel evolution within the animal kingdom. For example, the overall number of distinct genes from the given 12 genome set is estimated to be over 92,000, of which roughly 9% are shared among all genomes used in our study. The chart outlining the ratio of common, unique or taxon-/lineage- specific genes is given on Figure 2.

Our study estimates that the branch of the evolutionary tree leading to the chordate/human lineage has gained more than 10,000 genes from a common metazoan ancestor. Unlike the refinement proposed by Clamp et al. [12], our estimate does not exclude any ORFs with potentially non-coding or incomplete protein sequences and the set of genes found only in this branch maps to a smaller number of refined protein-coding genes. However, the estimated number of gained genes adequately reflects the degree of dissimilarity from the rough drafts of other distantly related genomes in this study. The chordate lineage represented by the human genome yields the highest number of unique genes, followed by *Daphnia *(12,647) also reflecting an enormous expansion of the gene complement within this rapidly evolving arthropod lineage. Of course, genes unique to a particular genome such as *Daphnia *or *Saccoglossus *might share a common ancestry with some other genes, but have deviated beyond recognition (i.e. in this case a BLAST e-value threshold). Adding more genomes is likely to enhance the resolution of gene gain and gene loss analysis.

**Figure 3:**
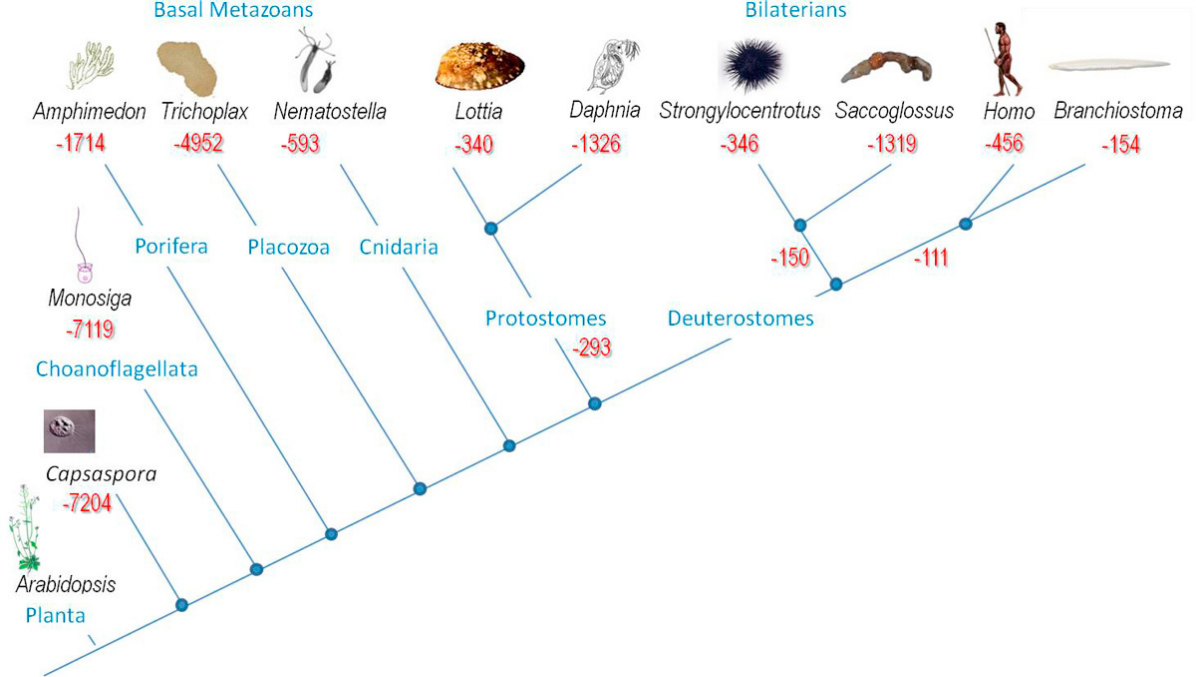
**Estimated gene loss in selected lineages within the animal kingdom (Metazoa)**.

Large degrees of disequilibrium between genes that were lost and gained in a particular branch may indicate trends towards increasing or decreasing morphological complexity. These situations are most apparent in Placozoa (*Trichoplax*), Choanoflagellates (*Monosiga*) and *Capsaspora *(all having more than 7,000 gene loss events). In contrast, *Amphimedon *(which might show a loss of only 1,700 genes from the common ancestor of all animals) and bilaterian genome composition in general revealed a lesser degree of gene loss. These numbers overlap with the predicted most likely position of the early branches off the root of the evolutionary tree of the animal kingdom (see Figure 3). Nevertheless, *Trichoplax *genome composition presents a special case and might be related to a significant secondary loss of morphological complexity and an overall gene complement from the common metazoan ancestor.

Comparison of genes shared in pairs of genomes exclusively is summarized in Table 3. While other nearest neighbors correspond to the nearest neighbors by traditional comparative morphology, *Trichoplax *shares an anomalously high number of putative gene homologs with a plant genome. Our analysis suggests a high degree of "contamination" of this *Trichoplax *genome with plant-like genes, possibly from symbiotic algae. Whether this influx results from genuine incorporation of horizontally transferred genes into host genomes or from a laboratory artifact remains to be seen.

**Table 3:** Anomalous number of gene families shared exclusively by Placozoan (tr) and Plant genomes (ar).

	am	ar	br	ca	da	ho	lo	mo	ne	pb	sa	tr	ur
am		0	24	9	63	13	18	0	83	4	2	0	13
ar			0	0	0	0	0	0	0	0	0	**10605**	0
br				6	30	124	77	0	102	3	60	0	42
ca					9	3	2	0	5	5	3	0	5
da						22	56	0	35	18	3	0	11
ho							29	0	30	3	12	0	28
lo								0	89	19	13	0	27
mo									0	9	0	0	0
ne										9	8	0	21
pb											0	2	6
sa												0	52
tr													0
ur													

Pair-wise table of genes found exclusively in two genomes. Abbreviations: am -- *Amphimedon*; ar -- *Arabidopsis*; br -- *Branchiostoma*; ca -- *Capsaspora*; da -- *Daphnia*; ho -- *Homo*; lo -- *Lottia*; mo -- *Monosiga*; ne -- *Nematostella*; pb -- *Pleurobrachia*; tr -- *Trichoplax*; ur -- Sea urchin (*Strongylocentrotus*).

The software is capable of comparing a few distantly related eukaryotic genomes using computational facilities available to a majority of academic research laboratories and in a time frame acceptable for research projects (under two weeks of computation in our case study). The most computationally demanding part is a reciprocal BLAST search, which scales well to the number of CPUs available. We produced tables of reciprocal BLAST results from the Linux machine at the Advanced Computing and Information Systems (ACIS) Lab, Electrical and Computer Engineering, University of Florida. The ASIC machine has 12 nodes (IBM x3850 M2 and X5) configured as four Non-Uniform Memory Access (NUMA), of which one was dedicated to our study. Each NUMA machine has 64 cores (Quad-core Xeon Tigerton 2.93 GHz and 8-core Xeon Nehalem 2.0 GHz) and 512 GB of Random Access Memory (RAM). Clustering, extraction and mapping of gene family occurrence in genomes has been done on a smaller desktop workstation with 16-core (2× 8-Core AMD Opteron 6136, 2400 MHz) and 32 GB RAM running Windows 7. The hard drive storage was not critical for purpose of the study and has not been described in detail; all initial setup, intermediate data and final results used a fraction of a 2TB partition of scrap space. The final output of our software could be a starting point for a number of branching research projects as the table of gene family occurrence in genomes can be interrogated in multiple ways with multiple logical expressions detailing macroevolution of distant taxa.

## Conclusion

The presented computational workflow allows the user to address specific questions regarding gene gain and gene loss in particular genomes and user-defined groups of genomes. We report a few interesting observations made using this new method and open source software implementation. For example, our analysis of 12 sequenced genomes indicated that these genomes possess at least 90,000 unique genes and gene families, suggesting enormous diversity of the genome repertoire in the animal kingdom. Approximately 9% of these gene families are shared universally (homologous) among all genomes, 53% are unique to specific taxa, and the rest are shared between two or more distantly related genomes. More results could be expected from analysis of gene gain and loss in distantly related phyla as new genomes are sequenced.

## List of abbreviations used

ACIS: Advanced Computing and Information Systems; BLAST: Basic Local Alignment Search Tool; COG: Cluster of Orthologous Genes; DAVID: The Database for Annotation, Visualization and Integrated Discovery; GO: Gene Ontology; KEGG: Kyoto Encyclopedia of Genes and Genomes; NUMA: Non-Uniform Memory Access; RAM: Random Access Memory.

## Competing interests

The authors declare that they have no competeting interests.

## Authors' contributions

LLM has provided the data, AP has designed and implemented the algorithms, AP and LLM have analyzed the data and written the manuscript.

## Supplementary Material

Additional file 1 Click here for file
